# Neuraxial-Enhanced Hemodynamic Control in a Nine-Year-Old Child With Pheochromocytoma: A Case Report

**DOI:** 10.7759/cureus.104224

**Published:** 2026-02-25

**Authors:** Rayan Muawad, Nezar M Alzughaibi, Khaled AlManea, Amal Al Farhan, Mohammed Alnamshan, Abdullah AlDhuwaihy, Mohammed E Alessa, Faisal Alomar

**Affiliations:** 1 Department of Pediatric Anesthesia, King Abdullah Specialist Children Hospital, Ministry of National Guard Health Affairs (MNGHA), Riyadh, SAU; 2 Division of Pediatric Surgery, Department of Surgery, King Abdullah Specialist Children Hospital, Ministry of National Guard Health Affairs (MNGHA), Riyadh, SAU; 3 College of Medicine, King Saud University, Riyadh, SAU

**Keywords:** hypertensive crisis, neuraxial anesthesia, pediatric-anesthesia, pediatric pheochromocytoma, perioperative management.

## Abstract

Pheochromocytoma is a rare cause of severe secondary hypertension in children, and its perioperative management can be particularly challenging due to catecholamine-mediated hemodynamic instability. We report a case of a nine-year-old male with glucose-6-phosphate dehydrogenase (G6PD) deficiency who presented with blurred vision and papilledema and was found to have a systolic blood pressure of 190 mmHg, consistent with a hypertensive emergency. Imaging revealed a 5.2 × 3.5 × 5.5 cm vascular left adrenal mass with central necrosis, and biochemical testing showed markedly elevated urinary vanillylmandelic acid and normetanephrines, confirming the diagnosis of adrenal pheochromocytoma. Echocardiography demonstrated mild to moderate concentric left ventricular hypertrophy.

Preoperative optimization included titrated alpha-adrenergic blockade with prazosin, sequential beta-blockade, and intravascular volume expansion. The anesthetic approach combined general anesthesia with a neuraxial enhanced technique, using intrathecal dexmedetomidine and morphine for sympatholysis, along with magnesium sulfate (50 mg·kg⁻¹) as a catecholamine-suppressing adjunct. Intraoperatively, only brief episodes of hypertension were observed, and these were effectively controlled with small boluses of labetalol and esmolol, without the need for continuous vasoactive infusions. Hemodynamics stabilized promptly after adrenal vein ligation. The postoperative course was uneventful. This report suggests that neuraxial enhanced multimodal sympatholytic strategies may facilitate stable perioperative hemodynamic control in pediatric pheochromocytoma.

## Introduction

Pheochromocytoma is a rare catecholamine-producing tumor of the adrenal medulla in children and is an important, though uncommon, cause of severe secondary hypertension [1]. Its typical pediatric presentations include sustained or paroxysmal hypertension with headache, sweating, and other adrenergic symptoms, which closely reflect the two-year history of episodic headache and profuse sweating reported in our patient [1]. Ocular manifestations such as visual disturbances, hypertensive retinopathy, or papilledema have been described in children with pheochromocytoma presenting in hypertensive crisis, consistent with this child’s initial presentation to the ophthalmology service with blurred vision and papilledema [2]. Hypertensive emergencies caused by pheochromocytoma can lead to hypertensive encephalopathy, stroke, and cardiac dysfunction, making early recognition and timely tumor resection critical for preventing irreversible end-organ injury [2].

The perioperative management of these patients remains challenging because anesthetic induction, airway manipulation, and tumor handling can precipitate abrupt catecholamine surges. At the same time, chronic norepinephrine-mediated vasoconstriction often leaves patients with contracted intravascular volume despite hypertension, thereby predisposing them to marked hemodynamic instability even with adequate preoperative preparation [3,4]. Standard practice emphasizes preoperative alpha-adrenergic blockade, cautious introduction of beta blockade, and intravascular volume expansion to reduce intraoperative blood pressure fluctuations and post-resection hypotension, all of which were applied in our patient [4].

In this report, we describe a case of a nine-year-old male with hypertensive emergency, papilledema, and biochemically confirmed adrenal Pheochromocytoma whose adrenalectomy was managed using optimized alpha-beta blockade, preoperative volume loading, magnesium sulfate, and a neuraxial-enhanced intrathecal dexmedetomidine-morphine strategy. Neuraxial-enhanced sympatholysis involves the targeted use of central alpha-2 agonists and opioids to blunt preganglionic sympathetic outflow during surgical stimulation. Although this approach carries potential risks, including bradycardia and hypotension, and the use of intrathecal dexmedetomidine in the pediatric population represents an off-label application, it was utilized in our case to achieve stable perioperative hemodynamics [5].

Written informed consent was obtained from the patient’s legal guardian.

## Case presentation

A nine-year-old male (weight 26.6 kg) with a known history of glucose-6-phosphate dehydrogenase (G6PD) deficiency presented to the ophthalmology clinic with blurred vision. Fundoscopic examination revealed papilledema, and he was referred to the emergency department, where he was found to have markedly elevated blood pressure, measuring 190/130 mmHg. He was admitted to a local pediatric ICU (PICU) with a diagnosis of hypertensive crisis and commenced on a continuous intravenous labetalol infusion. According to his mother, the patient had been experiencing recurrent, sudden-onset episodes of headache and profuse sweating for the preceding two years, with each episode lasting several minutes and resolving spontaneously without seeking medical care.

**Figure 1:**
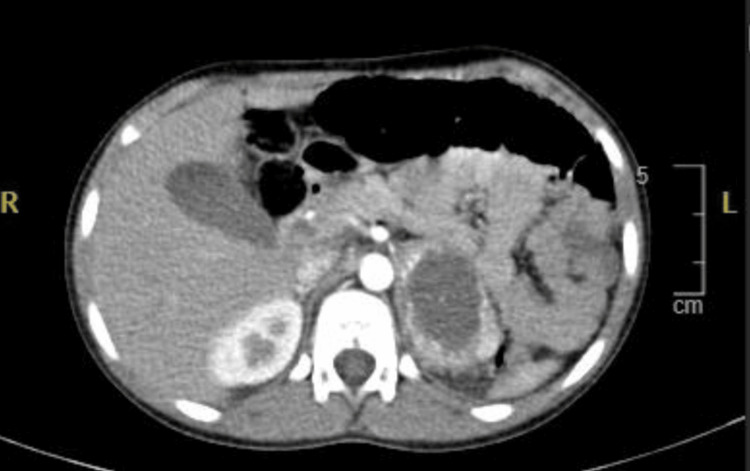
Axial contrast-enhanced abdominal CT scan demonstrating a large left adrenal pheochromocytoma with central necrosis CT: computed tomography

Cross-sectional imaging with contrast-enhanced CT of the abdomen and pelvis demonstrated a well-defined, oval-shaped left adrenal mass measuring 5.2 × 3.5 × 5.5 cm (anteroposterior × transverse × craniocaudal) (Figure 1). The lesion exhibited intense enhancement in the arterial phase, followed by progressive enhancement in the portal venous phase and contained a large central non-enhancing necrotic component measuring 4.2 × 2.7 × 3.6 cm, within which multiple hyperdense foci were identified, most consistent with vascular structures rather than calcifications. A small amount of adjacent retroperitoneal fluid accompanied by mild fat stranding was observed. The mass exerted a mild compressive effect on the superior pole of the left kidney, with preservation of the intervening fat plane and no radiological evidence of invasion, renal artery stenosis, or hydronephrosis. Several small retroperitoneal lymph nodes were noted, none of which met the criteria for pathological enlargement. The remaining abdominal organs appeared within normal limits. Brain imaging was unremarkable.

Transthoracic echocardiography performed before the transfer demonstrated mild-to-moderate concentric left ventricular hypertrophy with preserved systolic function, consistent with chronic pressure overload.

Upon arrival at our institution, the patient was hemodynamically stable on oral antihypertensive therapy and did not require further intravenous vasodilator support. He was alert, active, and in no distress. Cardiovascular examination revealed normal heart sounds without murmurs, well-perfused extremities, and a normal capillary refill time. The chest was clear to auscultation, and the abdomen was soft, non-distended, and non-tender, with no palpable masses. Neurological examination was non-focal. Continuous bedside monitoring over successive days demonstrated fluctuating yet gradually improving blood pressure control. Systolic blood pressure values generally ranged between 128 and 160 mmHg, and diastolic values between 80 and 98 mmHg, with heart rates of 90 to 125 beats per minute. Two brief episodes of severe hypertension accompanied by tachycardia were recorded; both responded promptly to rescue antihypertensive treatment and were not associated with neurological or cardiac complications.

Laboratory evaluation revealed a normal complete blood count and normal renal function. Serum electrolytes were within acceptable limits, with mild, clinically insignificant elevations in potassium and alanine aminotransferase that remained stable over time. Biochemical evaluation for catecholamine excess showed substantially elevated urinary catecholamine metabolites. The urinary vanillylmandelic acid (VMA)-to-creatinine ratio and random urinary normetanephrine-to-creatinine ratio were markedly increased, and a 24-hour urinary VMA collection was also significantly elevated. These findings, together with imaging and clinical presentation, confirmed the diagnosis of a catecholamine-secreting left adrenal pheochromocytoma.

A multidisciplinary team, including pediatric endocrinology, cardiology, nephrology, hematology, pediatric surgery, and pediatric anesthesiology, planned an elective left adrenalectomy after pharmacological optimization. The patient had been started on oral prazosin before transfer, which was gradually titrated to 4.5 mg three times daily under close monitoring. No complications from alpha-adrenergic blockade, such as symptomatic hypotension or reflex tachycardia, were noted. Intravenous hydralazine was prescribed as rescue therapy for breakthrough elevations in blood pressure. Preoperative targets included maintaining systolic blood pressure below 130 mmHg and heart rate below 100 beats per minute. Because sinus tachycardia persisted, a beta-adrenergic blocker was introduced, initially labetalol and later switched to atenolol (13.5 mg daily), while prazosin was continued as the primary alpha-1 antagonist.

In anticipation of intraoperative hemodynamic fluctuations and chronic catecholamine-induced vasoconstriction, intravenous maintenance fluids were increased to 1.5 times the standard rate the day before surgery to address relative intravascular volume depletion. On the day of surgery, prazosin and labetalol were administered, along with a single intravenous bolus of phentolamine 0.05 mg·kg⁻¹. In the preoperative holding area, the patient received intravenous midazolam 3 mg for anxiolysis. In the operating room, a loading dose of magnesium sulfate 50 mg·kg⁻¹ was given. General anesthesia was induced with propofol 100 mg, fentanyl 100 µg, and rocuronium 15 mg, followed by tracheal intubation and the establishment of invasive arterial and central venous monitoring. After induction, an enhanced neuraxial analgesic approach was implemented using a single-shot spinal injection of intrathecal dexmedetomidine 0.2 µg·kg⁻¹ and preservative-free morphine 5 µg·kg⁻¹ to provide analgesia and blunt catecholamine surges during tumor manipulation.

Baseline vitals before induction were approximately 128/91 mmHg with a heart rate of 114 beats per minute. Intraoperatively, the patient received both crystalloid and colloid fluids, including 5% albumin and lactated Ringer’s solution. A single bolus of intravenous labetalol 10 mg was given during early surgical dissection for transient hypertension. Tumor manipulation was associated with a brief episode of tachycardia and elevated blood pressure (peak 160/89 mmHg, heart rate 96 beats per minute), which responded promptly to a single bolus of esmolol 10 mg. Overall, hemodynamic fluctuations were minimal, and no continuous vasoactive infusions were necessary. Hemodynamic stability improved rapidly following adrenal vein ligation and tumor excision, with adequate urine output maintained throughout the procedure.

The total duration of surgery was approximately 3.5 hours. At the conclusion of the procedure, the patient was hemodynamically stable without vasoactive support and was transferred to the PICU for 24 hours of uneventful postoperative monitoring before being stepped down to the general pediatric ward.

## Discussion

The combination of long-standing, episodic headache and profuse sweating, accompanied by sudden severe hypertension and papilledema in our patient, is highly suggestive of catecholamine excess and aligns with previously reported pediatric pheochromocytoma cases presenting with headache, visual disturbances, and hypertensive crises [1]. Contrast-enhanced CT demonstrating a well-circumscribed, hypervascular left adrenal mass with central necrosis and no radiologic evidence of invasion corresponds closely to the typical imaging features of adrenal pheochromocytoma in children and supported proceeding with surgical adrenalectomy after medical optimization. Severely elevated urinary vanillylmandelic acid and normetanephrine levels confirmed the diagnosis of a catecholamine-secreting adrenal pheochromocytoma and are consistent with current guidelines recommending catecholamine metabolite testing as the biochemical standard for diagnosis [1].

Our preoperative regimen, with prazosin titrated to an effective alpha-blocking dose followed by the addition of beta blockade using atenolol and later labetalol, closely follows guidance that alpha-adrenergic antagonism should be established first, with beta blockers introduced only after adequate alpha blockade and continued up to the day of surgery [4]. Increasing maintenance intravenous fluids to 1.5 times the standard rate on the day before surgery aimed to correct catecholamine-induced intravascular volume depletion, in accordance with recommendations that preoperative volume expansion reduces the risk of severe post-resection hypotension and enhances intraoperative hemodynamic stability [4].

Magnesium sulfate was chosen as an intraoperative adjunct at 50 mg·kg⁻¹ because it suppresses catecholamine release, decreases alpha-adrenergic receptor sensitivity, and has been shown to aid hemodynamic control during pheochromocytoma surgery in both pediatric and adult patients [5]. More recent evidence from pediatric and adult cases suggests that magnesium is a versatile adjunct that helps maintain more stable blood pressure during pheochromocytoma surgery, consistent with the minimal hemodynamic fluctuations observed in our patient [6]. Although dexmedetomidine is most commonly administered intravenously, its ability as an alpha-2 agonist to reduce sympathetic outflow and stabilize blood pressure and heart rate during pheochromocytoma resection supported its use in our case as part of a neuraxial-enhanced intrathecal regimen combined with morphine to attenuate catecholamine surges during tumor manipulation [7].

Our decision to use the intrathecal route for dexmedetomidine was motivated by the need for targeted, site-specific sympatholysis to blunt the hemodynamic response to tumor manipulation. Additionally, we chose a single-shot spinal technique rather than a continuous epidural catheter to reduce the risk of prolonged postoperative hypotension. A recent study by Wiseman et al. identified epidural anesthesia as an independent risk factor for significant postoperative hypotension in pheochromocytoma resection (Hazard Ratio 3.49), likely due to sustained vasodilation after abrupt catecholamine withdrawal [8]. Local anesthetics administered via epidural or spinal routes produce a dense preganglionic sympathetic block. If the sympathetic nervous system is blocked at the time of adrenal vein ligation, the sudden drop in catecholamines eliminates compensatory vasoconstriction, resulting in severe vasoplegia. By avoiding local anesthetics entirely, our technique preserved sufficient intrinsic vascular tone to prevent profound hypotension. Furthermore, although intravenous dexmedetomidine infusion is a standard and effective alternative, we selected the intrathecal route to achieve dense, site-specific sympatholysis.

While evidence on pediatric neuraxial adjuvants is still emerging, a recent randomized trial by Fares et al. confirmed the safety and efficacy of intrathecal dexmedetomidine at 0.2 µg/kg in children undergoing major abdominal cancer surgery [9]. Notably, children receiving intrathecal dexmedetomidine maintained significantly lower intraoperative systolic blood pressure compared with those receiving fentanyl or bupivacaine alone [9]. In the context of pheochromocytoma, this predictable reduction in sympathetic tone, which might be viewed as an adverse effect in routine cases, provides a crucial therapeutic benefit by counteracting catecholamine-induced hypertensive crises. Additionally, the absence of respiratory depression or neurotoxicity in the pediatric cohort supports the safety of this neuraxial approach for complex pediatric procedures [9].

Case reports have described successful use of intravenous dexmedetomidine in adolescents and children undergoing adrenal pheochromocytoma resection, resulting in smoother intraoperative blood pressure and heart rate control and decreased need for vasodilators [6]. Taken together, this case contributes to the limited pediatric pheochromocytoma literature by showing that a neuraxial-enhanced approach that incorporates optimized alpha-beta blockade, preoperative volume expansion, magnesium loading, and intrathecal dexmedetomidine with morphine can achieve remarkably stable intraoperative hemodynamics in a child presenting with hypertensive emergency and papilledema [10]. However, given that current evidence for magnesium-dexmedetomidine and neuraxial-enhanced strategies in pediatric pheochromocytoma is largely limited to isolated case reports and small series, these approaches should be implemented only in experienced centers with invasive monitoring and immediate access to vasoactive medications, and further studies are required to establish their safety and generalizability [10].

## Conclusions

This report highlights that neuraxial-enhanced perioperative management can be safely incorporated into the anesthetic care of pediatric adrenal pheochromocytoma when combined with meticulous alpha-beta blockade and careful preoperative volume optimization. In a nine-year-old child presenting with hypertensive emergency and papilledema, the use of prazosin-based alpha blockade, sequential beta blockade, preoperative volume expansion, magnesium sulfate loading, and intrathecal dexmedetomidine with morphine was associated with remarkably stable intraoperative hemodynamics and eliminated the need for continuous vasoactive infusions. Our experience suggests that a multimodal sympatholytic strategy may enhance hemodynamic control during adrenalectomy for pediatric pheochromocytoma, particularly in high-risk cases with target-organ involvement. However, given that current evidence is limited to case reports and small series, neuraxial-enhanced strategies should be implemented only in centers with multidisciplinary expertise, invasive monitoring, and immediate access to vasoactive medications, and further research is required to define their safety, optimal dosing, and generalizability in children.
